# A randomized clinical trial of a peri-operative behavioral intervention to improve physical activity adherence and functional outcomes following total knee replacement

**DOI:** 10.1186/1471-2474-12-226

**Published:** 2011-10-07

**Authors:** Milagros C Rosal, David Ayers, Wenjun Li, Carol Oatis, Amy Borg, Hua Zheng, Patricia Franklin

**Affiliations:** 1 Department of Medicine, Division of Preventive and Behavioral Medicine, University of Massachusetts Medical School, 55 Lake Avenue North, Worcester, MA 01655, USA; 2 Department of Orthopedics and Physical Rehabilitation, University of Massachusetts Medical School, 55 Lake Avenue North, Worcester, MA 01655, USA; 3 Department of Physical Therapy, Arcadia University, 450 S. Easton Road, Glenside, PA 19038, USA

## Abstract

**Background:**

Total knee replacement (TKR) is a common and effective surgical procedure to relieve advanced knee arthritis that persists despite comprehensive medical treatment. Although TKR has excellent technical outcomes, significant variation in patient-reported functional improvement post-TKR exists. Evidence suggests that consistent post-TKR exercise and physical activity is associated with functional gain, and that this relationship is influenced by emotional health. The increasing use of TKR in the aging US population makes it critical to find strategies that maximize functional outcomes.

**Methods/Design:**

This randomized clinical trial (RCT) will test the efficacy of a theory-based telephone-delivered Patient Self-Management Support intervention that seeks to enhance adherence to independent exercise and activity among post- TKR patients. The intervention consists of 12 sessions, which begin prior to surgery and continue for approximately 9 weeks post-TKR. The intervention condition will be compared to a usual care control condition using a randomized design and a probabilistic sample of men and women. Assessments are conducted at baseline, eight weeks, and six- and twelve- months. The project is being conducted at a large healthcare system in Massachusetts. The study was designed to provide greater than 80% power for detecting a difference of 4 points in physical function (SF36/Physical Component Score) between conditions (standard deviation of 10) at six months with secondary outcomes collected at one year, assuming a loss to follow up rate of no more than 15%.

**Discussion:**

As TKR use expands, it is important to develop methods to identify patients at risk for sub-optimal functional outcome and to effectively intervene with the goal of optimizing functional outcomes. If shown efficacious, this peri-TKR intervention has the potential to change the paradigm for successful post-TKR care. We hypothesize that Patient Self-Management Support to enhance adherence to independent activity and exercise will enhance uniform, optimal improvement in post-TKR function and patient autonomy, the ultimate goals of TKR.

**Trial Registration:**

ClinicalTrials.gov: NCT00566826

## Background

The number of primary total knee replacement (TKR) procedures has grown by 73% in the past 10 years [1] and is projected to expand by more than 600% to 3.48 million procedures by 2030 [2]. Several facts are responsible for this increase in use, including (1) the prevalence of knee arthritis parallels the growing numbers of older adults, (2) increasing numbers of working aged adults are choosing TKR [1], and (3) TKR is a cost-effective intervention that reduces and eliminates pain. Despite these encouraging facts, significant variation remains in patient-reported functional improvement after TKR. An estimated 15-38% of patients report minimal functional improvement at 12 months, while another 10% report functional gains up to 3 times the national average [3,4]. Furthermore, our data suggests that the distribution of physical function at 12 months after TKR is bimodal [5]. The mechanism that underlies this varied functional improvement is not understood. While TKR researchers have identified some consistent predictors of poor gains in post-TKR function, no one patient attribute or surgical factor offers a satisfactory explanation for this outcome variation [6]. Importantly, poor emotional health as measured by the Mental Composite Score (MCS) of the SF36 is consistently associated with poor functional gain following TKR [3,4]. The NIH TKR Consensus Panel called for further research to address the role of patient attributes and rehabilitation regimens in functional outcome after TKR.

Exercise and activity improve global and knee-specific function in knee arthritis patients [7] and patient's confidence in their ability to manage their arthritis pain and function has been consistently associated with improved function, exercise, pain control, and self-care [8]. We have examined these associations in our pilot research and our data support a direct relationship between quantity of post-TKR exercise and activity and functional gain after TKR. This relationship was mediated by the patient's emotional health (SF36, MCS) and MCS was directly correlated with self-efficacy for arthritis self-care [9].

Based on the existing evidence, we hypothesize that a Patient Self-Management Support intervention that targets self-efficacy for physical activity and independent activity and exercise during the peri-operative TKR period will improve actual adherence to independent exercise and activity and, thus, maximize 6 and 12 month functional outcomes among post- TKR patients. This paper describes an ongoing study that is testing this hypothesis.

## Methods/Design

### Study Design

This prospective randomized clinical trial (RCT), funded by the National Institute for Arthritis and Musculoskeletal and Skin Diseases; NIAMS), is testing the efficacy of a theory-based telephone-delivered patient self-management support intervention to optimize post-TKR patient functional outcomes. The study participants are randomized to the Patient Self-Management Support intervention or to a usual care (control) condition, defined as care as prescribed by the patient's orthopedic surgeon or health care provider. The study seeks to assess the effect of the intervention on post TKR functional improvement, and understand the mechanisms of intervention effect by evaluating the roles of home exercise, physical activity, exercise self-efficacy, and participant attributes on functional improvement after TKR. It is hypothesized that, as compared to the control condition, patients receiving the Patient Self-Management Support intervention will have significantly greater increase in 6 and 12 month global (SF36/PCS) and knee-specific (WOMAC-Western Ontario and McMaster Universities Osteoarthritis Index) function. We also hypothesize that the intervention will enhance self-efficacy for exercise and that, in turn, increased self-efficacy will be associated with higher levels of home exercise and physical activity at 8 weeks and 6 and 12 month post-TKR. The study has approval from the Institutional Review Board of the Office of Human Subjects at the University of Massachusetts Medical School.

### Setting and population

The study is being implemented in collaboration with the Arthritis and Joint Replacement Center at UMass Memorial Medical Center (UMMHC), Worcester, MA, the sole dedicated arthroplasty center for the UMMHC 7-hospital system. Men and women 21 years of age or older and scheduled for primary, single-side TKR surgery undergoing surgery at the Arthritis and Joint Replacement Center are eligible for participation. Exclusions include: inflammatory arthritis, TKR due to fracture, malignancy, infection, or failure of a previous arthroplasty; inability to provide informed consent due to dementia or cognitive impairment; co-existing conditions that would negate functional improvement with surgery and exercise (i.e., severe Parkinson disease, or hemi paresis); emergently scheduled surgery; simultaneous bi-lateral TKR; terminal illness with life expectancy of less than one year; expected to be community dwelling after surgery; unavailable to complete the study procedures (i.e., will be out-of-region during rehabilitation period); and planning another TKR or total hip replacement surgery within 6 months.

### Recruitment, baseline assessment and randomization

The study coordinator recruits patients by reviewing a list of patients each week who are scheduled for upcoming TKR surgery, assessing patient's eligibility, calling patients to introduce the study and then mailing the consent form. The coordinator then follows up via telephone call. Patients complete and mail back a baseline assessment that includes the SF-36, the Pre-Surgery WOMAC, and a modified arthritis and exercise Self-Efficacy Scale. In addition, participants wear an ankle accelerometer (Step Activity Monitor) for four continuous days (at least one weekend day), and complete an exercise log.

Upon completing the baseline assessment, each patient is randomized into the Patient Self-Management Support intervention or to a usual care (control) condition. The study uses a stratified simple random sampling (S-SRS) of TKR patients to assure that participants represent subgroups of patients who may respond differently to the intervention. Patients are stratified by gender (two categories), BMI (three categories, < 30, 30-35, and > 35), and emotional health (SF36/Mental Composite Score; MCS) (three categories, < 45, 46-60, and > 60). This sampling scheme ensures that the sample includes wide variation in pre-operative BMI and MCS and gender balance. The categories are based on the distribution of pre-operative measures in a national sample. There are 2 × 3 × 3 = 18 strata in total. Within each stratum, 5 patients are randomized to intervention group and 5 to control group. The order of the two blocks is randomly permuted. Total target enrollment for the trial is 180 patients.

### Study conditions

#### Patient Self-Management Support intervention condition

##### Intervention theoretical framework

The intervention is based on social cognitive theory (SCT) [10]. Consistent with SCT, the intervention targets knowledge, attitudes (self-efficacy), and behavioral capabilities for self-management through a variety of literacy- and age-sensitive print materials and activities. The intervention sessions are delivered in accordance with the Patient-Centered Model [11-16], also known as the 5As model. This model guides the interventionist to deliver health behavior change counseling in a brief and structured manner. Accordingly, the counselor Asks about the patient's "*agenda*" for the encounter; *Assesses *the patient's experiences with specific behaviors (often milestone behaviors); *Advises *changes needed for improvement; *Assists *in setting goals for increasing activities and exercise and problem-solves with the individual potential challenges to goal attainment; and *Arranges *follow up. The counselor facilitates and reinforces self-efficacy for specific behaviors by progressively building on the patient's prior successes with the same or similar behaviors, assisting the patient in finding solutions for challenges encountered along the way, encouraging the patient to go a step further, and assisting the patient in eliciting the appropriate support from his or her social environment. Principles of motivational interviewing are used to facilitate the communication between the counselor and the patient [17]. This includes an emphasis on supportive (as opposed to confrontative) exchanges, exploring patient ambivalence and "rolling with resistance."

##### Intervention development

The intervention development was theory-based and its content was informed by thorough literature review of interventions for osteoarthritis arthritis, Internet-based health information, and interviews with TKR surgeons, hospital nurses, hospital- and community-based physical and occupational therapists, and interviews with patients. Challenges to successful post-TKR functional achievement were identified and strategies to address them were developed and integrated to form the intervention. Telephone counseling was selected as the primary basis for the intervention because it is a low-cost, broad reach mechanism for the delivery of post-TKR support interventions to patients with movement limitations and transportation difficulties. Although this counseling format has not been studied among TKR patients, it has been well studied for medical patients with other conditions and has been used alone or in combination with in-person sessions and print materials [18-20]. The initial intervention was further refined following a pre-test with 6 pilot patients.

##### Intervention Structure and Content

The final intervention protocol includes a total of 12 sessions: three prior to surgery, one call the evening before surgery, a hospital visit at 2-3 days after surgery, and seven additional sessions post-discharge between weeks two and nine post-surgery. All sessions, except for the hospital visit are telephone-based. The first call is approximately 45 minutes on average, with follow up contacts ranging from 5 to 20 minutes.

**Figure 1:**
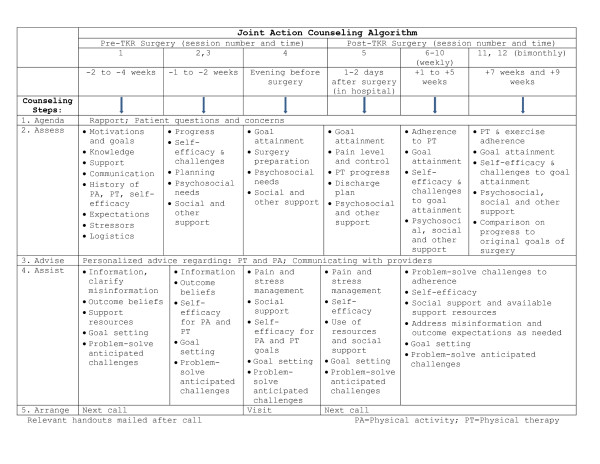
**Counseling Algorithm Enhancing Functional Outcomes of Patients Undergoing Total Knee Replacement Surgery**.

The intervention aims to build self-management skills through the implementation of behavioral strategies. In accordance with the 5As model described above, at each contact the coach and the patient discuss a clear *Agenda*, and the coach *Assesses *knowledge needs, attitudes toward surgery or rehabilitation process (i.e., self-efficacy), and patients' physical activity and exercise behaviors. The counselor delivers brief personalized *Advice, Assists *patients in identifying exercise and physical activity goals and creating an action plan, and *Arranges *follow up. All patients receive the following print materials: benchmarks for recovery, tips for regaining the use of the knee, goal-setting and monitoring forms, and tips on setting goals. Additional intervention materials include a "Toolkit" of one-page print materials that the coach can mail to participants to reinforce strategies or address patient questions or concerns. Pre-surgery coaching topics include: communicating with health care providers and making the most of medical appointments, asking for help from family and friends, where to go after leaving the hospital, tips for choosing a rehabilitation center, sleeping well away from home, understanding the risk of infection, and tips to tracking exercises by using an exercise log. Hospital-related topics include: managing pain in the hospital, help with pain after the hospital, fear of pain medication, conquering constipation, using a continuous passive motion (CPM) machine, breathing well in the hospital and what to do if having trouble breathing, things that can get in the way of recovery, taking care of the incision and scar, helping with swelling, and benefits of ongoing physical activity. Post-surgery topics include: walking again, using a stationary bicycle, physical activity at home, ongoing physical activity, activities that may be limited after the surgery, using exercise and activity logs, joining local groups for physical activity, eating for recovery, and how to tell if making progress. Topics related to emotional health include: tips to help with anxiety, depression, fatigue, and stress. The intervention is delivered in English and Spanish by a trained bilingual health educator. Figure 1 depicts components of the intervention protocol over time.

##### Intervention fidelity

Several procedures are implemented to maximize intervention fidelity. The intervention coach was trained in the delivery of the intervention protocol by the investigators. A customized Microsoft Access database guides the delivery of specific intervention components (5A's, as described above). Calls are audiotaped for random review by the behavioral researcher who provides timely feedback to the coach concerning errors of omission or commission, and additional training as needed.

##### Participant safety

A Safety Monitoring Plan is followed. All adverse events are first reported to the PI, the lead surgeon, and the lead behavioral scientist for appropriate action. All serious adverse events (such as hospitalizations), and other adverse events (depression) are reported to the Safety Officer and to the sponsor, NIAMS. Patients who express significant depressive symptoms either to the study Coach or study coordinator, or have a CES-D score ≥ 16, are encouraged to discuss their feelings with their health care provider and to seek professional help. The study team (physical therapist, psychologist and PI) provides consultation and supervision on medical, rehabilitative and psychosocial issues to the coach throughout the program.

#### Control Condition

The control condition consists of usual care as prescribed by the participant's health care providers, including surgical management and physical therapy. Patients in the control condition also receive the copy of standard post-TKR exercises and one goal planning sheet. The surgeon and treating physical therapist are blinded to the study condition and do not participate in data collection of study end-points. However, it is possible for patients to mention the study coach calls to the surgeon at a follow-up visit.

#### Study assessments

Data are collected at baseline prior to surgery and at 8 weeks, 6 months, and 12 months post-TKR. Sources of data include: (1) patients, (2) surgical records, (3) physical therapy records, and (4) data recorded by the intervention coach in the intervention database. The following section describes individual measures used to assess outcomes of interest.

#### Main outcomes

##### Physical Function

The SF-36 Version 2, a generic 36 item assessment of health status is used to assess physical (PCS) and mental (MCS) function [21-24].

##### Knee Function

WOMAC, a 17 item survey, is used to assess knee-specific measures of the degree of difficulty in performing daily activities (e.g., descending stairs) plus joint-specific pain and stiffness [25].

#### Secondary outcomes

Knee performance measures include timed up and go (TUG), timed stairs, and 6 minute walk [26,27]. Knee impairment measures include knee range of motion (ROM) and measurements of knee flexion and extension ROM [28,29]. These measures are collected by trained physical therapists with demonstrated clinically acceptable reliability.

#### Mediators

##### Self-Efficacy

The Stanford Patient Education Research Center Arthritis Self-Efficacy tool was modified slightly to address the post-surgical experience [30]. Scale items assess an individual's perceived ability to manage pain/discomfort and frustration, and keep knee pain from interfering with roles and activities.

##### Exercise and Activity

A *Step Activity Monitor *(accelerometer) assesses mean steps per day and activity intensity (steps per minute). The monitor provides a summary of total number of steps/cycle per 24 hour period and percent time/day that the individual is active at high/moderate/low activity, % of day inactive, and maximum sustained intensity for 5 and 30 minutes [31]. In addition, participants complete an *exercise (paper) log *to record the number of daily exercise sessions, repetitions and sets for each of the core home-based physical therapy exercises. The log is completed at 8 weeks and 6 months.

#### Potential moderators

In addition to the above measures, the study assesses participants' demographic and psychosocial characteristics (i.e., age, gender, residence/zip code, race/ethnicity, educational level, employment, insurance status, depressive symptoms [32], trait anxiety [33], physical co-morbid conditions [34], social support [35,36], and body mass index (BMI).

#### Sample size considerations and statistical analyses

##### Sample size estimation

Sample sizes were calculated using the method developed by Frison and Pocock [37] with the SF-36 PCS as the primary outcome. Because patients are randomized within surgeon, we assume complete randomization of the study subjects to each treatment arm. We used the mean changes of repeated measures to estimate the required sample sizes based on Analysis of Covariance (ANCOVA) method. The mean change is defined as the difference between each subject's mean post-treatment PCS (the mean of 6 and 12 month PCS) and the baseline measurement.

##### Effect Size

This trial was designed to determine whether the intervention will outperform the control by 4 or more points in the SF36 PCS. This effect size is approximately one-half of the PCS standard deviation and 40% of the mean pre-post PCS TKR change of 10.5 points reported in the PORT study. Based on our pilot data and the national registry of TKR patients, mean post-PCS scores are assumed to be 39.5 and 43.5 for the control and intervention arms, respectively; the standard deviation of post-TKR PCS is estimated as 10.9 for pre-and post-intervention periods and the autocorrelation coefficient between the post treatment measurements across time for PCS is 0.65, and between pre-and post treatment measures is 0.50 [4]. One pre-intervention and two post-intervention measurements were completed (6 and 12 months). Assuming 75 completers per condition after attrition or exclusion due to missing data, the trial is capable of detecting an intervention effect of a change of 4 or more points on the SF36 PCS with statistical power in excess of 0.83 at two-sided significance level of 0.05, or 0.90 at one-sided significance level of 0.05. Secondarily, a 10 point difference in change in WOMAC physical functional scores between the arms can be detected with > 85% power at 5% significance level.

##### Analysis Plan

We will test the self-care support program's effect, both overall and in the latent low and high function response groups, as predicted by baseline characteristics on post-TKR 6 and 12 month functional outcome. We will apply mixture models with two latent classes (or GLLAMM), where the post-operative change in global function (PCS/SF36) at 6 or12 months is the primary outcome. The main predictors of the model include intervention indicator (0 = control versus 1 = intervention), data collection time points, and the interaction term between intervention indicator and time points. The treatment effects will be evaluated by testing the significance of the coefficient of intervention indicator and an interaction term of time and the intervention indicator. In the analysis, we will adjust for other patient characteristics that may moderate the intervention effect or reduce unexplained variability including gender, age, baseline function score, BMI, depression, anxiety, or social support. Potential moderating effects of each variable on the intervention will be examined by including interaction terms with the intervention indicator. The intervention effects on changes in knee pain, function using WOMAC sub-scores, number of daily repetitions and mean steps/hour on step activity monitor, and self-efficacy scores) will be analyzed using similar approaches. If the proposed intervention is effective, the post-TKR increases in home exercise and activity levels, and self-efficacy score in the intervention condition should be significantly greater than in the control condition. Next, we will examine whether the improvement in Stanford Arthritis Self-Efficacy scores predicts increases in home exercise and activity levels. Using a similar approach, we will test whether higher levels of home exercise (number of daily repetitions) and physical activity (mean steps/day on accelerometer) at 8 weeks post-TKR are associated with greater improvement in 6 and 12 month post-TKR global physical function and knee function.

In the analysis, we will assess possible selection bias by evaluating the equivalence in subjects' socio-demographic characteristics and baseline clinical and behavioral measures between "completers" (i.e., patients participating in 6 and/or 12 month follow-up) and drop-outs. If systematic and significant differences are observed, we will apply proper statistical procedures to account for such informative attrition or missingness. (See below) Differences between completers and drop-outs will be acknowledged as limitations to the generalization of study results.

##### Self-care Program Cost Analysis

An estimate of the annual cost of developing and refining the self-care program will be calculated from the perspective of the surgeon's office at one point in time. Intervention program logs will be used to estimate the recurring labor and operational costs of delivering the program. Because the research team will have delivered the program to 90 patients, we will calculate the telephone counselor time, phone costs, and print material costs per patient required to prepare and deliver telephone and in-hospital sessions. In addition, we will estimate the cost of behavioral expert supervision. Sensitivity analyses will test the range of assumptions for telephone counselor time and salary, print material costs, and clerical scheduling time. This secondary analysis will serve future dissemination discussions.

## Conclusion

This paper presented the theoretical rationale and design of an ongoing randomized controlled trial that will test the efficacy of a Patient Self-Management Support intervention on TKR functional outcomes. TKR is a common and well accepted intervention for advanced knee arthritis and, thus, maximizing functional outcomes is of utmost priority. If efficacious, this intervention has considerable potential for dissemination. In addition to the potential for patient support programs to enhance self-management, lessons learned in this trial may inform tailored post-TKR rehabilitation strategies, including home activity and exercise regimens.

## Competing interests

The authors declare that they have no competing interests.

## Authors' contributions

MCR, DA, WL and PF contributed to the study design. MCR, CO, DA and PF designed the intervention and oversee the study implementation. HZ, AB and PF designed the data collection system to support the intervention and integrate patient assessments. All authors read and approved the final manuscript.

## Pre-publication history

The pre-publication history for this paper can be accessed here:

http://www.biomedcentral.com/1471-2474/12/226/prepub
